# Long-term effects of pancreas transplant alone on nephropathy in type 1 diabetic patients with optimal renal function

**DOI:** 10.1371/journal.pone.0191421

**Published:** 2018-01-29

**Authors:** Sung Shin, Chang Hee Jung, Ji Yoon Choi, Hyun Wook Kwon, Joo Hee Jung, Young Hoon Kim, Duck Jong Han

**Affiliations:** 1 Division of Kidney and Pancreas Transplantation, Department of Surgery, Asan Medical Center, University of Ulsan College of Medicine, Seoul, Korea; 2 Asan Diabetes Center, University of Ulsan College of Medicine, Seoul, Republic of Korea; Icahn School of Medicine at Mount Sinai, UNITED STATES

## Abstract

**Background:**

Limited data are available regarding optimal selection criteria for pancreas transplant alone (PTA) to minimize aggravation of diabetic nephropathy.

**Methods:**

A total of 87 type 1 diabetic patients were evaluated before and after PTA at a single center from January, 1999 to December, 2015, together with 87 matched non-transplanted type 1 diabetic subjects who were candidates for PTA to compare deterioration of native kidney function. A total of 163 patients (79 in the transplanted group and 84 in the nontransplanted group) were finally enrolled after excluding nine patients with estimated glomerular filtration rate less than 60 mL/min/1.73 m^2^ and two patients with moderate proteinuria (≥ 1.5 g/day).

**Results:**

A total of seven recipients (8.9%) had end-stage renal disease post-transplant whereas only one patient (1.2%) developed end-stage renal disease in the nontransplanted group during their follow-up period (median 12.0, range 6–96 months) (p = 0.03). Furthermore, a composite of severe renal dysfunction and end-stage renal disease (31.6% vs 2.4%) was significantly higher in the transplanted group (p < 0.001). Multivariate Cox regression analysis revealed that a higher level of tacrolimus at six months post-transplant (HR = 1.648, CI = 1.140–2.385, p = 0.008) was the only significant factor associated with end-stage renal disease.

**Conclusions:**

There is a considerable risk for deterioration of renal function in PTA recipients post-transplant compared with non-transplant diabetic patients. With rather strict selection criteria such as preoperative proteinuria and estimated glomerular filtration rate, PTA should be considered in diabetic patients to minimize post-transplant aggravation of diabetic nephropathy.

## Introduction

It is known that pancreas transplantation can restore insulin-independence in patients with type 1 diabetes mellitus (T1DM) [1–3]. Several studies have reported an increase in the long term pancreas graft survival rate which has been attributed to the new developments in immunosuppressants and advances in surgical techniques [4–7].

Previous studies reported that diabetic nephropathy improved in nonuremic, type 1 diabetic patients who had pancreas transplant alone (PTA) [8–10]. Although it has been known that PTA recipients have the potential benefits on the course of diabetic nephropathy, severe renal dysfunction and end-stage renal disease have been reported in some PTA recipients [11–13]. It has been previously reported that pre-PTA renal dysfunction, age, gender, and persistent high trough levels of tacrolimus were contributing factors for ESRD after PTA [2, 13, 14].

Therefore, it is still uncertain whether the native kidney is more severely deteriorated after PTA compared with nontransplanted type 1 diabetic patients. The aim of this study is to compare the progression of diabetic nephropathy between PTA recipients and nontransplanted type 1 diabetic patients and to identify risk factors for severe dysfunction or ESRD after PTA.

## Research design and methods

### Patients and procedures

This is an observational cohort study using data on recipients of PTA at our center. ESRD events were verified from the electronic medical records of our center. ESRD was defined as the need for being on dialysis or kidney transplantation. Severe renal dysfunction was defined as a decrease of estimated glomerular filtration rate (eGFR) more than 50% compared with that pre-transplant or at the beginning of observation. The eGFR was measured by the Chronic Kidney Disease Epidemiology Collaboration (CKD-EPI) formula. CKD-EPI eGFR was estimated both as continuous and categorical variables. The latter was constructed by classifying eGFR into two clinically meaningful groups (i.e., ≥ 90, and ≥ 60 and < 90 mL/min/1.73 m^2^). This study was performed after receiving approval from the Institutional Review Board of Asan Medical Center (S2017-1438-0001). The need for consent of participants was waived by the ethics committee because this is a retrospective study without any diagnostic or therapeutic intervention. T1DM patients who underwent PTA at our center were retrospectively investigated with prospectively collected data. Indications for PTA were the presence of one or more overt diabetic complications and/or glucose hyperlability with hypoglycemic unawareness and impaired quality of life [15–17].

Surgical procedures of PTA were performed and postoperative anticoagulation was administered as previously described [18]. On retrieval of a pancreas graft, the abdominal organs were perfused through the aorta with Histidine-tryptophan-ketoglutarate (HTK; 10–15 L). The graft portal vein was anastomosed end-to-side to the recipient’s external iliac vein. The superior mesenteric and splenic arteries reconstructed by donor iliac arterial Y-graft were anastomosed to the recipient’s common iliac or external iliac artery. Drainage of the exocrine pancreatic secretions was performed by bladder in all the recipients. Patients were administered continuous intravenous heparin (400–1000 U/hr). The level of activated partial thromboplastin time (aPTT) was checked every 6 hours, after which they were administered oral warfarin for three months. The target level of aPTT and PT (international normalized ratio) was maintained at 1.5 to 2 of the upper reference range. All patients underwent CT angiography for monitoring vascular patency within 48 hours following pancreas transplantation.

### Immunosuppressions

The main immunosuppression protocol consisted of antithymocyte globulin (ATG) induction, maintenance with tacrolimus and mycophenolate mofetil (MMF), and steroids. The total ATG dose was 4.5–5.0 mg/kg in all the PTA recipients. The first dose (1.5 mg/kg) was intraoperatively administered and followed by 1 mg/kg ATG on postoperative days 1, 2, 4, and 6. A target tacrolimus level of 8–10 μg/L was achieved within seven days in 90% of all of the patients. Seven of the 79 recipients received basiliximab (Simulect) as induction therapy. Rejection was defined using clinical parameters and biopsies. Treatment for T cell-mediated rejection included pulse steroids or ATG whereas total plasma exchange, intravenous immunoglobulin, and rituximab were considered for antibody-mediated rejection.

### Statistical analysis

Categorical variables are expressed in terms of the absolute and relative frequencies. Quantitative variables are expressed as the mean and standard deviation (SD). Differences between means were analyzed using the Student’s t test or the Mann-Whitney U test, as appropriate. Categorical variables were compared using the chi-square test. Logistic regression was used to model the association of pre-transplant estimated glomerular filtration rate (eGFR) and proteinuria with the risk of ESRD or severe renal dysfunction (> 50% decrease) after PTA. Univariable and multivariable analyses were performed for a time-to-event analysis, using the Cox proportional hazard model, to verify significant factors for ESRD or severe renal dysfunction after PTA. Death-censored graft survival was analyzed using the Kaplan-Meier estimator and log-rank tests.

## Results

### Demographic and baseline characteristics

Between January, 1999 and December, 2015, PTA was performed in a total of 91 patients. Among them, four patients with type 2 diabetes were excluded from our study. Among the 87 transplanted type 1 diabetic patients, 79 recipients were enrolled in the final analysis after excluding eight with eGFR less than 60 mL/min/1.73 m^2^. Of 275 nontransplanted type 1 diabetic patients who were candidates for PTA, 87 subjects were matched according to age, sex, and the duration of diabetes. Among them, three patients were excluded due to moderate proteinuria (> 1.5g/day) or eGFR less than 60 mL/min/1.73 m^2^. As shown in Table 1, there was no significant difference between the two groups in baseline characteristics except that those in the transplanted group were significantly younger, the onset of diabetes was significantly earlier in the transplanted group, and the mean follow-up period was significantly longer in the nontransplanted group. There was no significant difference in initial proteinuria (82.7±22.4 mg/day vs 82.6±20.7 mg/day, p = 0.999). There was no patient with initial eGFR less than 60 mL/min/1.73 m^2^.

**Table 1:** Baseline characteristics of type 1 diabetic patients enrolled in the study.

Variables	Nontransplanted group (n = 84)	Transplanted group (n = 79)	p-value
Mean age, y (SD)	31.6 (10.0)	28.5 (9.1)	0.042
Female gender, n (%)	51 (60.7)	47 (59.5)	1.000
Body mass index, kg/m^2^ (SD)	22.1 (3.3)	21.2 (2.8)	0.072
Onset of diabetes, y (SD)	23.1 (9.1)	18.6 (9.9)	0.003
Duration of diabetes, y (SD)	8.9 (7.2)	9.8 (6.5)	0.421
Insulin dose, IU/day (SD)	39.8 (16.0)	43.5 (21.2)	0.209
Neuropathy, n (%)	7 (8.3)	14 (17.7)	0.101
Retinopahty, n (%)	26 (31.0)	31 (39.2)	0.325
Hypertension, n (%)	5 (6.0)	3 (3.8)	0.721
Dyslipidemia, n (%)	8 (9.5)	3 (3.8)	0.213
Initial proteinuria, mg/day (SD)	82.7 (22.4)	82.6 (20.7)	0.999
Follow-up period, months (SD)	83.7 (28.5)	41.3 (35.8)	< 0.001

The characteristics of the recipient and donor at baseline, as well as the transplant parameters are described in Table 2 according to the pre-transplant eGFR (CKD-EPI, mL/min/1.73 m^2^). Among the 79 recipients, 23 (29.1%) had a pre-transplant eGFR between 60 and 90 mL/min/1.73 m^2^ and 56 (70.9%) had a pre-transplant eGFR >90 mL/min/1.73 m^2^. There was no significant difference between the two groups.

**Table 2:** Baseline characteristics of PTA^a^ recipients according to pre-transplant eGFR^b^ (mL/min/1.73 m2) (CKD-EPI^c^).

Variables	60 ≤ eGFR < 90 (n = 23)	eGFR ≥ 90 (n = 56)	p-value
**Recipient characteristics**			
Mean age, y (SD)	30.0 (10.0)	27.9 (8.7)	0.338
Female gender, n (%)	13 (56.5)	34 (60.7)	0.803
Body mass index, kg/m^2^ (SD)	21.7 (3.1)	21.0 (2.7)	0.302
Onset of diabetes, y (SD)	18.7 (10.8)	18.5 (9.7)	0.940
Duration of diabetes, y (SD)	11.3 (7.7)	9.2 (6.0)	0.168
Presensitized patients (PRA^d^ > 20%), n (%)	2 (8.7)	6 (10.7)	1.000
HLA-DR mismatch, n (SD)	1.5 (0.7)	1.2 (0.7)	0.106
Donor characteristics			
Mean age, y (SD)	25.2 (9.5)	27.5 (10.0)	0.340
Female gender, n (%)	6 (26.1)	24 (47.1)	0.125
Body mass index, kg/m2 (SD)	21.5 (2.8)	21.4 (3.2)	0.967
Cold ischemia time, (SD)	5.2 (1.7)	5.8 (2.3)	0.299
Cause of death, CVA^e^, n (%)	3 (13.0)	5 (8.9)	0.685
Immunosuppressants and others			
Induction Regimen, n (%)			0.097
Rabbit Anti-thymocyte globulin	23 (100)	49 (87.5)	
Interleukin-2 receptor blocker	0	4 (7.1)	
Daclizumab	0	3 (5.4)	
Calcineurin inhibitor, n (%)			0.552
Tacrolimus	23 (100)	53 (94.6)	
Cyclosporine	0	3 (5.4)	

^a^Pancreas transplant alone

^b^Estimated glomerular filtration rate

^c^Chronic kidney disease epidemiology

^d^Panel reactive antibody

^e^Cerebrovascular accident

### Comparison of the metabolic variables between transplanted group and nontransplanted group

**Fig 1:**
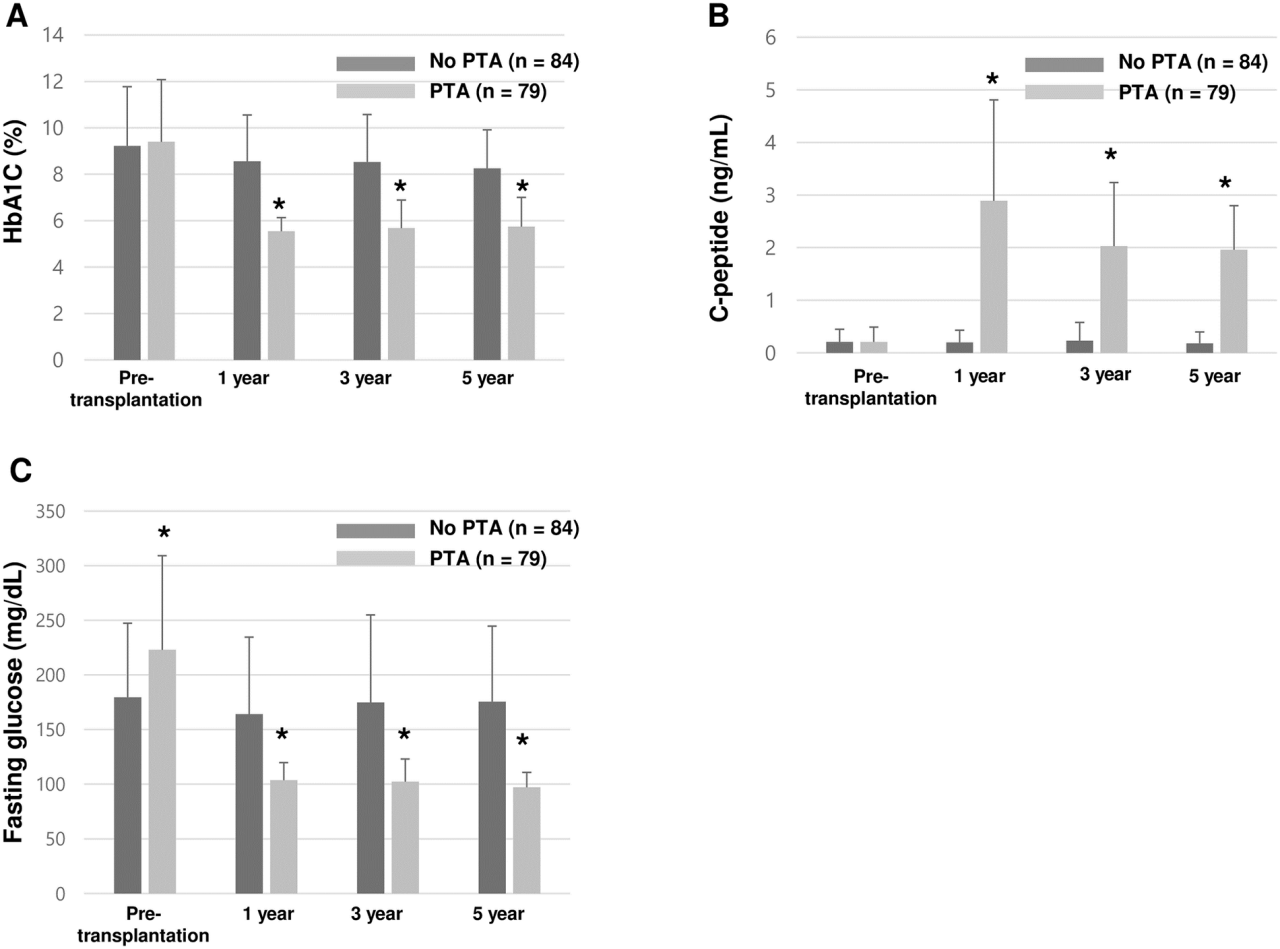
Comparison of the mean level of HbA1c (A), C-peptide (B), and fasting glucose (C) between nontransplanted type 1 diabetic patients and PTA recipients during the five-year follow-up period. * p < 0.001.

There was no significant difference in the level of HbA1c between the nontransplanted group and the PTA recipients before pancreas transplantation (9.2±2.5% vs 9.4±2.7%, p = 0.677) (Fig 1A). However, the mean HbA1c levels in the PTA recipients had kept constant below 6.0% while the mean HbA1c levels in the nontransplanted group was significantly higher compared with those in the PTA recipients during the five-year follow-up period (Fig 1A). There was no significant difference in the level of C-peptide between the nontransplanted group and the PTA recipients before pancreas transplantation (0.21±0.24ng/mL vs 0.21±0.28ng/mL, p = 0.898) (Fig 1B). The postoperative mean levels of C-peptide in the PTA recipients were significantly higher than in the nontransplanted group during the five-year follow-up period (Fig 1B). However, the levels decreased steadily and which was confirmed by a linear mixed effect model (p < 0.001) (Fig 1B). Although preoperative levels of fasting glucose in the PTA group were significantly higher compared with those in the nontransplanted group (p < 0.001), the mean fasting glucose levels in the PTA group remained lower than in the nontransplanted group throughout the five-year follow-up period (Fig 1C).

### Comparison of native kidney failure and severe dysfunction between transplanted group and nontransplanted group

**Fig 2:**
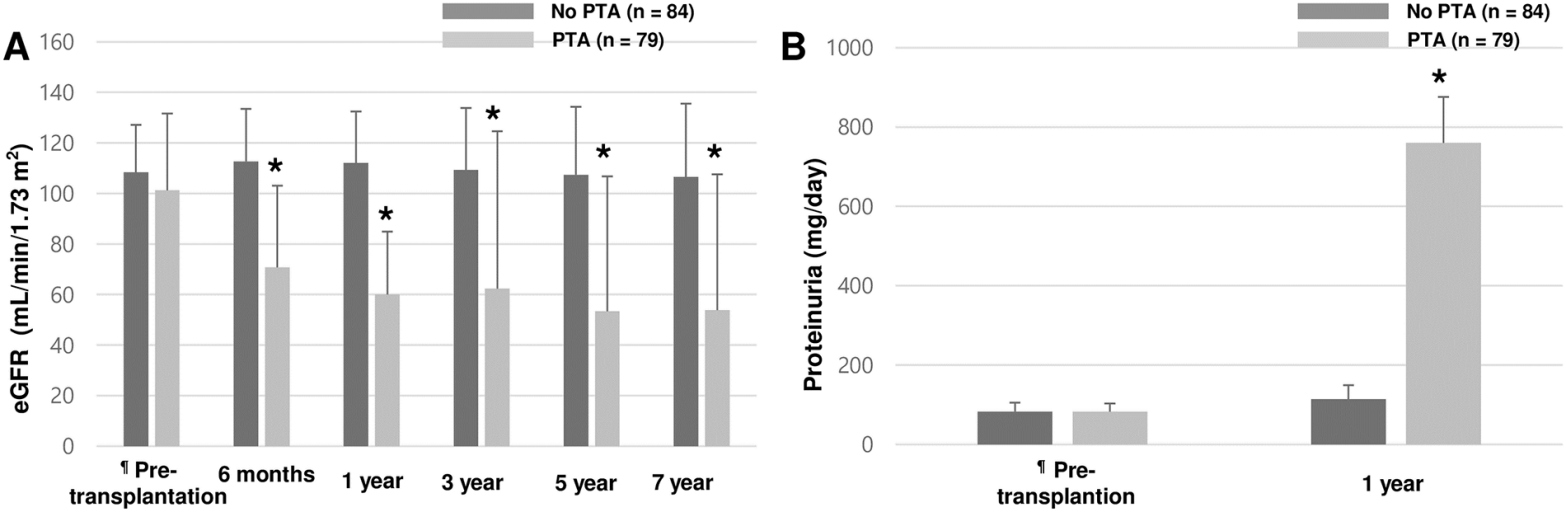
Comparison of the mean level of eGFR (A) and proteinuria for 24 hours (B) between nontransplanted type 1 diabetic patients and PTA recipients during the follow-up periods. ^¶^ Pre-transplantation for PTA recipients and beginning of observation for nontransplanted patients; * p < 0.001.

**Fig 3:**
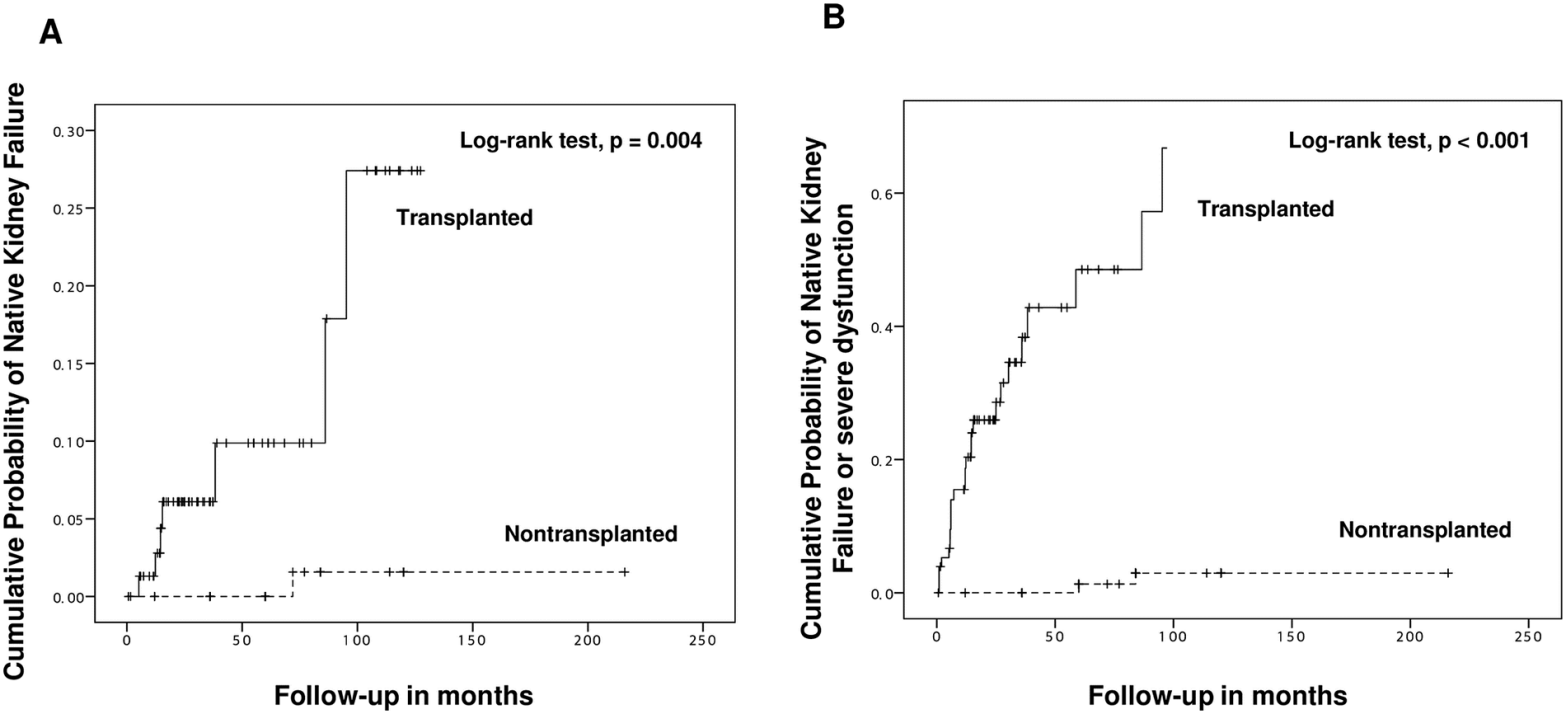
Cumulative probability of (A) native kidney failure and (B) a composite outcome of native kidney failure and severe dysfunction.

There was no significant difference in eGFR at pre-transplantation period (or at the beginning of observation) between the two groups (101.2±30.4 mL/min/1.73 m^2^ vs 108.4 mL/min/1.73 m^2^, p = 0.063). During the seven-year follow-up period, however, the mean level of eGFR in the PTA group was significantly higher compared with those in the nontransplanted group (Fig 2A). Furthermore, the mean level of 24 hour-proteinuria at one year post-transplant (759.9±115.9mg/day vs 114.3±35.2mg/day, p < 0.001) was significantly higher in the PTA group compared with those in the nontransplanted group while there was no significant difference at pre-transplantation period (or at the beginning of observation) (82.6±20.7mg/day vs 82.7±22.4mg/day, p = 0.999) (Fig 2B). A total of seven PTA recipients (8.9%) had ESRD post-transplant whereas only one patient (1.2%) developed ESRD in the nontransplanted group during their follow-up period (p = 0.03). A cumulative incidence of ESRD in the transplanted group was significantly higher than those in the nontransplanted group (Fig 3A). Furthermore, a composite of severe renal dysfunction and ESRD (31.6% vs 2.4%, p < 0.001) was significantly higher in the transplanted group (Fig 3B). In addition, ESRD or severe renal dysfunction (21.0±25.3 months vs 72.0±17.0 months, p = 0.01) in the transplanted group was developed relatively in early posttransplant period compared with those in the nontransplanted group.

### Risk factors for native kidney failure and severe dysfunction after PTA

By univariate analysis, we found that PTA recipients who developed ESRD had higher preoperative proteinuria, a higher level of preoperative hemoglobin A1c, the use of cyclosporine rather than tacrolimus, a higher trough level of calcineurin inhibitor at six months post-transplant, readmission due to metabolic acidosis (Table 3). Multivariate analysis revealed that posttransplant ESRD was found to be associated only with a higher level of calcineurin inhibitor at six months posttransplant (OR, 1.368; 95% CI, 1.023–1.829; p = 0.034).

**Table 3:** The risk of end-stage renal disease after pancreas transplant alone and adjusted HR from multivariate cox regression.

Variables	HR_unadj_^a^	HR_adj_^b^	95% CI^c^	p-value
Preoperative proteinuria	1.003	1.002	0.998–1.005	0.308
Preoperative hemoglobin A1c	1.286	1.308	0.948–1.805	0.102
Cyclosporine (vs. Tacrolimus)	9.640	1.029	0.076–13.899	0.983
Trough level of CNI^d^ at six months post-transplant	1.294	1.368	1.023–1.829	0.034
Readmission due to metabolic acidosis	5.788	5.747	0.639–51.651	0.119

^a^ Hazard rate unadjusted

^b^ Hazard rate adjusted

^c^ Confidence interval

^d^ Calcineurin inhibitor

In addition, the risk of a composite outcome of ESRD and severe renal dysfunction was validated in the PTA recipients. By univariate analysis, a higher level of calcineurin inhibitor at six months posttransplant, preoperative neuropathy, and preoperative retinopathy was associated with the composite outcome. By multivariate analysis, however, there was no independent factor associated with the composite outcome.

A total of eighteen patients (22.8%) had pancreas graft failure during the follow-up period (median 11.9 months, range 4–56 months). There was a significant difference in neither eGFR nor 24 hour-proteinuria during the follow-up period between those with and without pancreas graft failure (Data not shown). On the other hand, a total of fifteen recipients (20.3%) developed acute rejection during the follow-up period (median 6.0 months, range 1–38 months). There were five cases of heavy venous thrombus post-transplant which necessitated surgical or interventional thrombectomy while there were two cases of leakage at anastomosis for exocrine drainage. Neither of them aggravated renal function in the follow-up period. In addition, there was no significant correlation between the mean levels of C-peptide post-transplant and renal function in terms of eGFR and 24 hour-proteinuria (S1 Fig).

### Impacts of preoperative eGFR and proteinuria on long-term native kidney survival and function

**Fig 4:**
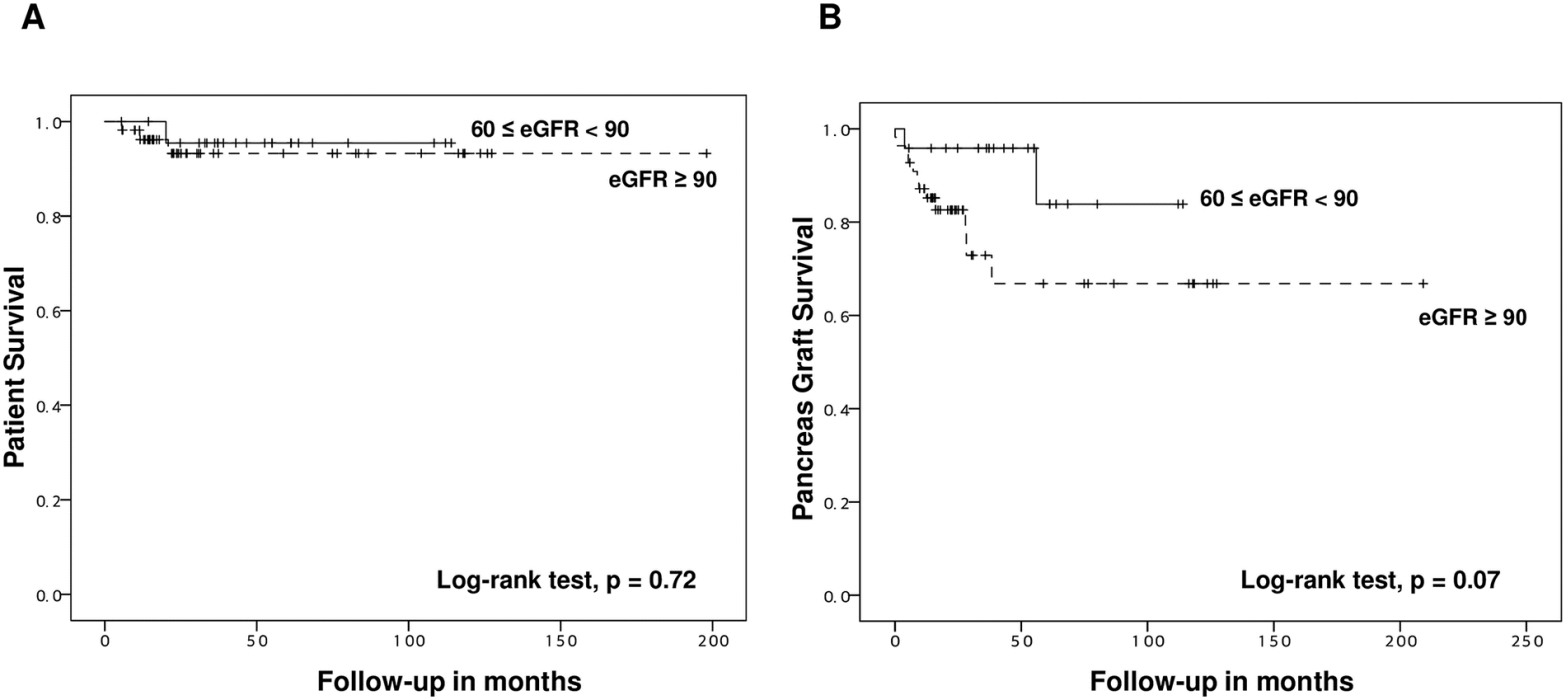
Kaplan-Meier curves for more than 15-year (A) patient survival and (B) death-censored pancreas allograft survival according to a preoperative eGFR.

**Fig 5:**
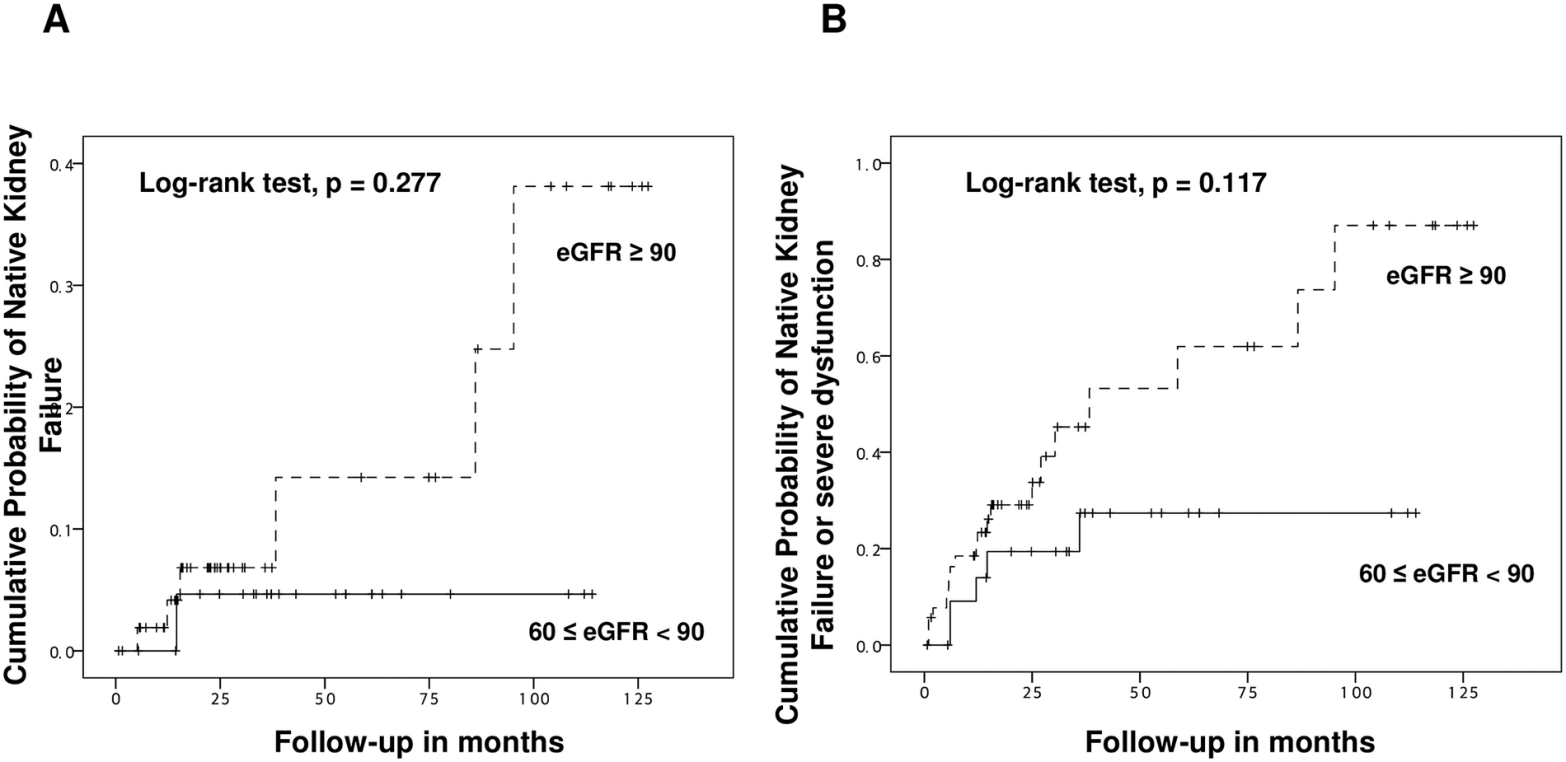
Kaplan-Meier curves for 10-year cumulative probability of native kidney failure (A) and a composite outcome of native kidney failure and severe dysfunction (B) according to a preoperative eGFR.

Kaplan-Meier curves for more than 15-year revealed that there was a significant difference in neither mortality of PTA recipients nor death-censored pancreas graft survival among two groups according to preoperative eGFR (Fig 4A and 4B). Meanwhile, there was a significant difference in neither cumulative probability of native kidney failure (Fig 5A), nor cumulative probability of a composite outcome of native kidney failure and severe dysfunction (Fig 5B) between the two groups. However, even though a preoperative eGFR was more than 90mL/min/1.73 m^2^, the risk of native kidney failure or severe dysfunction was considerable.

## Discussion

In this study, we found that there was a considerable long-term risk of ESRD or severe renal dysfunction after PTA in type 1 diabetic recipients compared with non-transplant type 1 diabetic patients although PTA recipients gained the long-term improvement of endocrine function.

Several literatures have been published which evaluated beneficial versus detrimental effects of PTA on diabetic nephropathy in type 1 diabetic patients as mentioned earlier [8–13]. Coppelli et al. reported that successful PTA determines improvement of diabetic nephropathy as documented by a significant reduction of average urinary excretion rate and regression of proteinuria in several patients 1 year after transplantation [9]. They reported that creatinine concentrations and clearances did not differ before and after transplantation. This study is different from theirs in that we validated the long-term effects of PTA on diabetic nephropathy. Boggi et al. verified the long-term efficacy and safety of PTA in type 1 diabetic patients [8]. They found a significant decrease in the level of proteinuria after transplantation whereas a 20% cumulative decrease of GFR over the five years of follow-up. They suggested that the decrease of GFR in eight PTA recipients was less than type 1 diabetic patients on the waiting list for islet transplantation. In this study, however, ESRD or severe renal dysfunction in the transplanted group was developed relatively in early posttransplant period compared with those in the matched nontransplanted group even though they had optimal renal function before PTA. Furthermore, two (28.6%) PTA recipients developed ESRD within one year posttransplant while none in the nontransplanted group developed ESRD over the five years of follow-up. Although a few studies demonstrated that kidney function deteriorated significantly one year after PTA [12, 19], they did not present long-term outcomes regarding the deterioration of native renal function after PTA.

Successful pancreas transplantation has been shown to stabilize or reverse the histologic abnormalities of diabetic nephropathy in native kidneys when normoglycemia is maintained for five to ten years [20, 21]. However, this study showed that the native kidneys in not a few recipients may be severely damaged in the early period. Various factors seemed to have been involved in the deterioration of native kidney function after PTA. Posttransplant ESRD was found to be associated with a higher level of calcineurin inhibitor at six months posttransplant. It has been reported that a lower preoperative eGFR was associated with a greater likelihood of progression to clinically significant kidney disease [22–24]. Of note, even though a preoperative eGFR was more than 90mL/min/1.73 m^2^, there was a considerable risk of native kidney failure after PTA in our analysis. It was also reported that a higher risk of native kidney failure among recipients who underwent treatment for rejection episodes of pancreas graft [2, 19]. It is postulated that a higher level of calcineurin inhibitor might be accepted when a PTA recipient experienced one or more episodes of rejection aggravating diabetic nephropathy. It was reported that tacrolimus levels at six months postoperatively higher than the target levels was the only parameter identified as an independent prognostic factor for the development of substantial decline in native renal function [14]. Proteinuria prior to PTA reflects the underlying subclinical diabetic nephropathy. Therefore, it should be considered that pre-PTA heavy proteinuria may predispose the native kidney to irreversible injury even to ESRD post-PTA. It might be reasonable that type 1 diabetic patients who have heavy proteinuria or eGFR less than 60 should have simultaneous kidney and pancreas (SPK) transplantation. In South Korea, however, a chance for allocation of a SPK is given only to candidates who are on dialysis. No matter how native kidneys are severely deteriorated, they cannot get a chance for a SPK unless they are on dialysis according to regulations established by Korean Network for Organ Sharing. In the United States, candidates can be listed for an isolated kidney or combined kidney/pancreas transplant if they meet the minimum criteria of (a) measured or calculated creatinine clearance or glomerular filtration rate (Cockcroft-Gault or other reliable formula) equal to and less than 20 mL/min; or (b) initiation of maintenance dialysis [25]. However, geographical, ethical and center differences in listing and transplantation performance have been reported in adult patients with preemptive registration, mainly in the United Kingdom [26, 27] and in the United States [28, 29]. It is worthy to be considered that type 1 diabetic patients with deteriorating native kidneys get priority for a SPK even if they are not on dialysis yet.

In conclusion, despite the retrospective, single-center, observational nature of this study, the present study demonstrates a long-term deteriorating effect of PTA on native kidney function and identifies contributing factors for ESRD or severe renal dysfunction. It is suggested that type 1 diabetic patients with borderline renal function or heavy proteinuria need to be consulted for the possibility of the native kidney failure when they consider PTA. Although PTA recipients gained the long-term improvement of endocrine function, they are exposed to native kidney injuries associated with a higher level of calcineurin inhibitor.

## Supporting information

S1 File Raw data of this study (SPSS). This file contains raw data about clinical variables as well as metabolic variables of each patient in this study.
(SAV)
